# Deep-Brain Subthalamic Nucleus Stimulation Enhances Food-Related Motivation by Influencing Neuroinflammation and Anxiety Levels in a Rat Model of Early-Stage Parkinson’s Disease

**DOI:** 10.3390/ijms242316916

**Published:** 2023-11-29

**Authors:** Beata Grembecka, Irena Majkutewicz, Oliwia Harackiewicz, Danuta Wrona

**Affiliations:** Department of Animal and Human Physiology, Faculty of Biology, University of Gdańsk, Wita Stwosza 59, 80-308 Gdańsk, Poland; irena.majkutewicz@ug.edu.pl (I.M.); oliwia.harackiewicz@phdstud.ug.edu.pl (O.H.); danuta.lewandowska@ug.edu.pl (D.W.)

**Keywords:** subthalamic nucleus deep-brain stimulation, Parkinson’s disease, 6-hydroxydopamine, interleukin-6, cFos protein, lymphocytes, corticosterone, food-related motivation, anxiety

## Abstract

Deep-brain subthalamic nucleus stimulation (DBS-STN) has become a well-established therapeutic option for advanced Parkinson’s disease (PD). While the motor benefits of DBS-STN are widely acknowledged, the neuropsychiatric effects are still being investigated. Beyond its immediate effects on neuronal circuits, emerging research suggests that DBS-STN might also modulate the peripheral inflammation and neuroinflammation. In this work, we assessed the effects of DBS-STN on food-related motivation, food intake pattern, and the level of anxiety and compared them with markers of cellular and immune activation in nigrostriatal and mesolimbic areas in rats with the 6-OHDA model of early PD. To evaluate the potential mechanism of observed effects, we also measured corticosterone concentration in plasma and leukocyte distribution in peripheral blood. We found that DBS-STN applied during neurodegeneration has beneficial effects on food intake pattern and motivation and reduces anxiety. These behavioral effects occur with reduced percentages of IL-6-labeled cells in the ventral tegmental area and substantia nigra pars compacta in the stimulated brain hemisphere. At the same brain structures, the cFos cell activations were confirmed. Simultaneously, the corticosterone plasma concentration was elevated, and the peripheral blood lymphocytes were reduced after DBS-STN. We believe that comprehending the relationship between the effects of DBS-STN on inflammation and its therapeutic results is essential for optimizing DBS therapy in PD.

## 1. Introduction

The deep-brain stimulation of the subthalamic nucleus (DBS-STN) is an effective and widely used treatment for motor symptoms in the late stage of Parkinson’s disease (PD) [1]. DBS-STN is a safe procedure, and numerous studies have demonstrated that this surgical treatment can significantly enhance the motor skills of patients with PD [2]. In addition to motor benefits, some studies have indicated the influence of DBS-STN on emotional symptoms and cognitive function [2,3]. DBS-STN in PD patients improves depression and anxiety scores [4] and influences motivation [5]. Although the motor effects of DBS-STN for PD patients are spectacular, the mechanism of DBS-STN action still remains uncertain. Research indicates that the effects observed after DBS-STN in PD patients may be related to the role of the STN in the mesolimbic system [3]. The STN is involved in processing information in the basal-ganglia–limbic network [5,6,7]. In rats, the STN sends afferent projections to the ventral tegmental area (VTA)—the main region of dopaminergic mesolimbic and the hypothalamus—the main center for the regulation of food intake and energy metabolism [8,9]. The traditional role of the STN in the basal ganglia network places it within motor circuits. The modulatory properties of DBS-STN [10] appear to be important for the emotional effects of this method in PD patients; however, the mechanisms underlying these changes are still controversial.

DBS-STN has been shown to increase cell firing in structures innervated by the STN [11]. Microdialysis studies have confirmed that glutamate release in output basal ganglia structures is increased after DBS-STN [12]. In addition, there is accumulating evidence that DBS-STN increases striatal dopamine efflux and metabolism in rats [13,14]. Norepinephrine and dopamine are neurotransmitters that play roles in reward processing and motivation, and their abnormal serum levels have been reported in depressed rodents [15]. The clinical observations of PD patients after DBS-STN reported craving for sweet food in some cases or decreased addictive behavior toward dopaminergic treatment [16]. The animal study documented the opposite effect of DBS-STN on the cocaine-induced and natural reward and proved that DBS-STN reduces craving for cocaine while increasing craving for sucrose [17]. The other study showed that lesions of STN decreased incentive motivation (seeking behavior) for cocaine while inducing the opposite effect (facilitating incentive motivation) for food [18,19,20]. On the other hand, many studies have reported increases in apathy after DBS-STN (for revival, see [21]), defined as a loss of motivation or reduction in goal-directed behavior. One explanation for the emergence or worsening of apathy following DBS-STN is a withdrawal-like syndrome due to the reduction of dopaminergic treatment [22,23]. There is also evidence supporting the role of DBS-STN itself in this pathogenesis [24,25]. It is difficult to determine the underlying causes of motivational symptoms arising after DBS-STN in the clinic due to the interaction between DBS-STN, pharmacological co-treatments, and the progression of PD pathology. It seems that the emotional effect after DBS-STN may be linked to hormonal imbalance and weight gain, which are reported after DBS-STN in clinical cases [26,27]. Studies suggest that dysregulated hypothalamic–pituitary–adrenal axis (HPA) activity is linked to an overproduction of pro-inflammatory cytokines including tumor necrosis factor alpha (TNF-α), interleukin (IL)-6, and IL-1β. This, in turn, contributes to the impairment of hippocampal neurogenesis and the promotion of depression-like behaviors [28,29]. 

Dissociating the effects of DBS-STN itself from underlying neuropathology and co-occurring pharmacological treatment is critical to understand DBS-STN’s influence on emotion [22]. The objective of this study was to enhance our understanding of the anti-inflammatory properties of DBS-STN in a rodent model of Parkinson’s disease. The anti-inflammatory effects of the administered stimulation were associated with the evaluation of motivation and anxiety levels, behaviors that undergo changes as a result of DBS-STN application in PD patients. In this manner, we aimed to identify a potential mechanism responsible for some emotional effects observed after DBS-STN. Our previous research showed that DBS-STN applied in rats with advanced models of PD elevated corticosterone levels and influenced the peripheral numbers of lymphocytes [30]. A previous study performed by our research team showed also that the electrical or pharmacological activation of the VTA influences peripheral blood immunity, feeding behavior, and neuronal activation in the hypothalamus [31,32]. Since pro-inflammatory activity is hypothesized to be already present in the prodromal stages of PD [33] and DBS-STN is considered to be a potential anti-inflammatory method [30,34], explaining the mechanism of DBS-STN seems important for its use in PD patients. To study the effects of DBS-STN applied in partially nigral-depleted Wistar male rats on neuronal activation and neuroinflammation, we measured IL-6 and cFos expression in the dopaminergic structures of the nigriostriatal (substantia nigra pars compacta, SNpc) and mesolimbic (VTA) systems. To link changes in the brain expression of IL-6 and cFos after DBS-STN with behavior, we studied the food intake patterns and motivations (Vermicelli handling test, VHT) and the levels of anxiety (elevated plus maze, EPM). The peripheral immune system activation during neurodegeneration was also measured using peripheral blood morphology and corticosterone levels. Thus, understanding the role of DBS-STN on emotional processing and its anti-inflammatory properties may contribute to defining DBS-STN’s mechanism of action in PD patients.

## 2. Results

### 2.1. PD Model and Electrode Placement Confirmation

**Figure 1:**
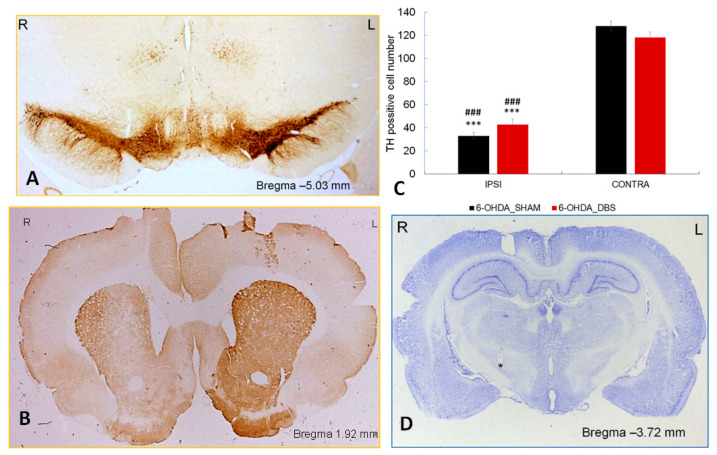
Verification of 6-OHDA lesion (**A**,**B**) and electrode placement (**D**). Photomicrographs (representative rat from 6-OHDA_DBS group) of substantia nigra (**A**) and striatum (**B**)—TH-immunostained sections from rat sacrificed 7 days after DBS-STN, applied on the next day after 6-OHDA nigral injection (L—left, R—right hemisphere), and tip (asterisk) of stimulating electrode located in right STN (**D**). Graph (**C**) shows the number of TH+ cells in the substantia nigra pars compacta, counted using stereology in 6-OHDA_DBS (*n* = 18) and 6-OHDA_SHAM (*n* = 18) rats in IPSI (right) and CONTRA—lateral hemisphere to the DBS-STN. Data are represented as means ± SEM. *** *p* < 0.001 vs. 6-OHDA_SHAM CONTRA; ### *p* < 0.001 vs. 6-OHDA_DBS CONTRA, Tukey HSD after ANOVA.

The parkinsonian phenotype was confirmed in 6-OHDA lesioned rats using behavioral VHT testing (described below) and TH quantification (Figure 1). The intranigral administration of 6-OHDA at the right brain hemisphere induced a reduction of TH-immunoreactive neurons in the SNpc and its terminals in the striatum, as demonstrated by immunohistochemistry performed 7 days after neurotoxin injection (Figure 1A,B). The quantification of TH+ neurons revealed a significant (F (3.72) = 128.55; *p* < 0.001, one-way ANOVA) reduction in the number of TH-positive body cells in the right SNpc of 6-OHDA-depleted rats in both DBS-STN-stimulated (6-OHDA_DBS) and control (6-OHDA_SHAM) animals. The quantification of the number of labeled cells in the SNpc on the lesioned side was reduced by 42% (*p* < 0.001, Tukey post-hoc) in 6-OHDA_SHAM and by 35% (*p* < 0.001, Tukey post-hoc) in 6-OHDA_DBS (F (3.72) = 128.55) in comparison to the non-lesioned (contralateral) hemisphere (Figure 1C). The Nissl staining showed that the localization of the tips of stimulating electrodes was in the STN region between 3.60 and 3.80 mm posterior to the bregma (Figure 1D).

### 2.2. DBS-STN Effects on Motivation for Food and Contralateral Forepaw Impairment

**Figure 2:**
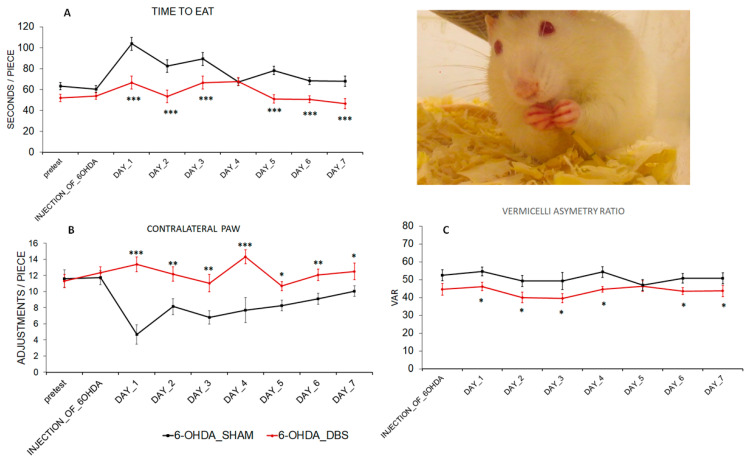
Effect of DBS-STN on food intake pattern and food intake motivation (Vermicelli handling test) in rats with early-stage PD. (**A**) Time to eat a single pasta piece as a determinant of motivation for food in rats before PD model induction (injection_of_6-OHDA) and after subsequent days (DAY_1–DAY_7) of DBS-STN (6-OHDA_DBS, *n* = 6) or SHAM (6-OHDA_SHAM, *n* = 6) stimulation. (**B**,**C**). Contralateral paw adjustment during pasta eating and Vermicelli asymmetry ratio (VAR) as a determinant of food intake pattern in the same experimental condition. Data are means ± SEM. Explanation: * *p* < 0.05, ** *p* < 0.01, *** *p* < 0.001 differences vs. 6_OHDA_SHAM (Kruskal–Wallis test, post-hoc Dunn).

The Kruskal–Wallis test revealed that 6-OHDA_DBS rats have significantly shorter eating times for a single piece of pasta from day 1 to 3 and from day 5 to 7 of the stimulation procedure (DAY_1 to DAY_3 and DAY_5 to DAY_7) in comparison to the 6-OHDA_SHAM group [DAY_1 (H = 15.84; *p* ≤ 0.001), DAY_2 (H = 12.75; *p* ≤ 0.001), DAY_3 (H = 6.86; *p* ≤ 0.001), DAY_5 (H = 22.97; *p* ≤ 0.001), DAY_6 (H = 16.49; *p* ≤ 0.001), DAY_7 (H = 20.50; *p* ≤ 0.001)] (Figure 2A). The Kruskal–Wallis test confirmed, also, that the number of contralateral paw adjustments increased after each day of DBS_STN (DAY_1 (H = 24.85 *p* ≤ 0.001), DAY_2 (H = 6.93 *p* ≤ 0.01), DAY_3 (H = 7.53 *p* ≤ 0.01), DAY_4 (H = 11.27; *p* ≤ 0.001), DAY_5 (H = 6.39; *p* ≤ 0.05; DAY_6 (H = 6.74 *p* ≤ 0.01), DAY_7 (H = 4.79 *p* ≤ 0.05), Figure 2B). In parallel with increased contralateral paw efficiency, the VAR was reduced in 6-OHDA_DBS rats (DAY_1 (H = 3. 83; *p* ≤ 0.01), DAY_2 (H = 4.55; *p* ≤ 0.01), DAY_3 (H = 5.03; *p* ≤ 0.01), DAY_5 (H = 3.21; *p* ≤ 0.05), DAY_6 (H = 3.30; *p* ≤ 0.01), DAY_7 (H = 5.52; *p* ≤ 0.01), Figure 2C), which was substantiated through Kruskal–Wallis testing.

### 2.3. DBS-STN Affects Anxiety Level

**Figure 3:**
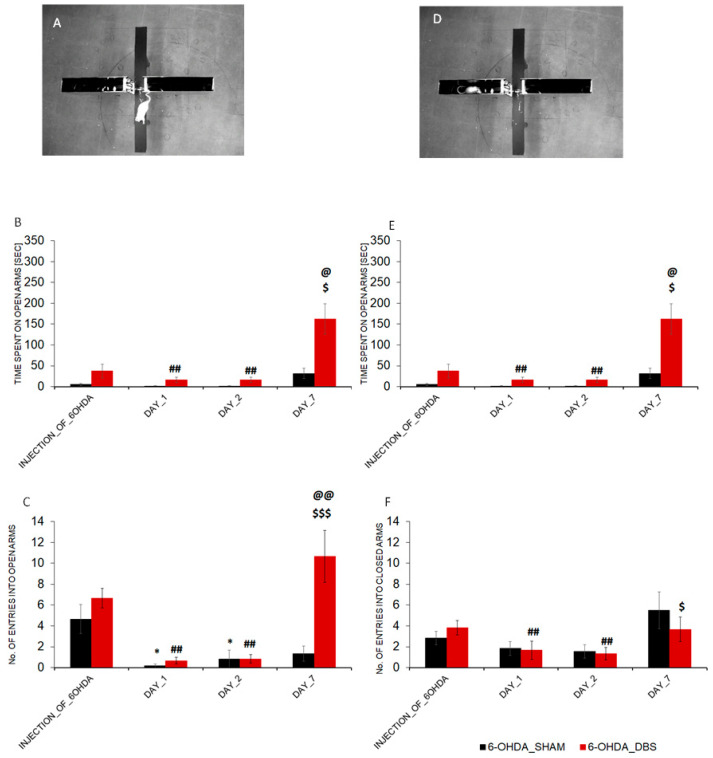
Effect of DBS-STN on anxiety-like behaviors in rats with early-stage PD. (**A**,**D**) EPM test conditions during exploration of open or closed arms of maze. (**B**,**E**) Number of entries into the open or closed arms of maze before PD model induction (injection_of_6-OHDA) and after one (DAY_1), two (DAY_2), or seven days (DAY_7) of DBS-STN (6-OHDA_DBS, *n* = 6) or SHAM (6-OHDA_SHAM, *n* = 6). (**C**,**F**) The cumulative durations of time spent in open or closed arms of EPM, measured at this same point of procedure in both groups. Data are means ± SEM. Explanation: * *p* < 0.05 differences vs. baseline (injection_of_6-OHDA) for 6_OHDA_SHAM; ## *p* < 0.01 differences vs. baseline (injection_of_6-OHDA) for 6_OHDA_DBS; @ *p* < 0.05, @@ *p* < 0.01 differences vs. 6-OHDA_SHAM; $ *p* < 0.05, $$$ *p* < 0.01 differences vs. DAY_1 of DBS-STN procedure applied in 6-OHDA_DBS (Kruskal–Wallis test and post-hoc Dunn test).

The Kruskal–Wallis test revealed that rats after DBS-STN have had been significantly affected in terms of the total number of open-arm entries (H = 31.179, *p* < 0.001) and number of closed-arm entries (H = 16.062, *p* < 0.05) and time spent in each of the arms (H = 27.537, *p* < 0.001 and H = 14.173, *p* < 0.05) in comparison to 6-OHDA_SHAM animals. The Dunn test confirmed that all 6-OHDA lesioned rats showed an increased level of anxiety after 6-OHDA injection (DAY_1 and DAY_2) compared to the respective group baseline conditions (injection of 6-OHDA; Figure 3C). The reduced numbers of entries on the open arm of EPM in 6-OHDA_SHAM animals (*p* < 0.05 and *p* < 0.05) and 6-OHDA_DBS animals (*p* < 0.01 and *p* < 0.01) were confirmed using Dunn post-hoc tests. At the same points of the procedure, the time spent on open arms was reduced only in 6-OHDA_DBS rats (*p* < 0.01 and *p* < 0.01, Dunn post-hoc). The anxiolytic-like behaviors were observed in DAY_7 of DBS_STN and were reflected by a higher number of entries and time spent in open arms for 6-OHDA_DBS rats in comparison to 6-OHDA_SHAM rats (*p* < 0.01 and *p* < 0.05, Dunn post-hoc) and in comparison to DAY_1 (*p* < 0.001 and *p* < 0.05; Dunn post-hoc, Figure 3B,C). The numbers of entries in closed arms of EPM in DAY_1 and DAY_2 were affected in both the 6-OHDA and lesioned groups, but only in 6-OHDA_DBS, the means were statistically significant in comparison to the respective baseline (*p* < 0.01 and *p* < 0.01; Dunn post-hoc, Figure 3E). On the last day of the procedure (DAY_7), 6-OHDA-depleted rats without DBS-STN chose the closed arms of EPM more often than 6-OHDA_DBS rats (*p* < 0.05; Dunn post-hoc, Figure 3E). The time spent in closed arms of EPM was reduced in both 6-OHDA-depleted groups after 7 days of procedure in comparison to the baseline (injection of 6-OHDA) (*p* < 0.001 and *p* < 0.05; Dunn post-hoc, Figure 3F).

### 2.4. DBS-STN Influence on Percentage of cFos+ Cells and 6-OHDA Induced-Changes in the Percentage of IL-6+ and Double-Labeled Cells (cFos+/IL-6)

**Figure 4:**
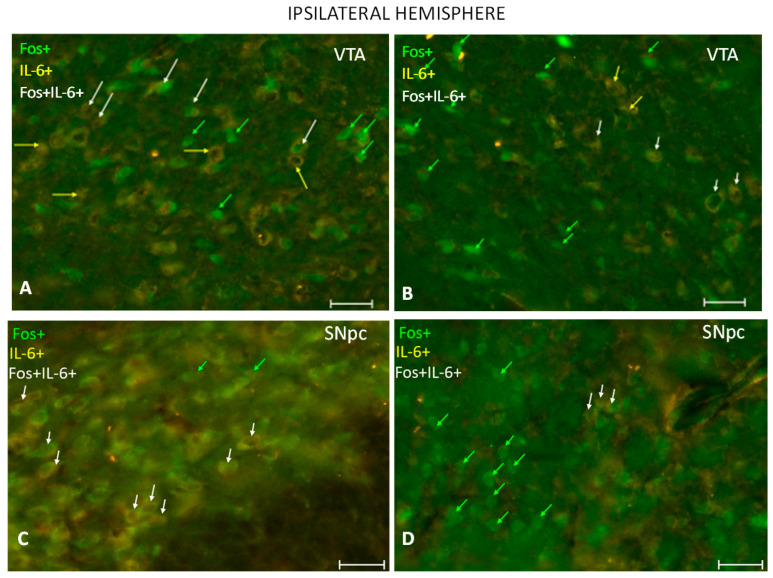

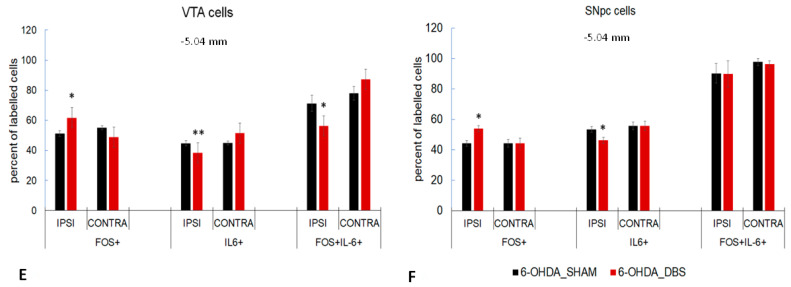
Effect of DBS-STN on neuroinflammation and cell activation in dopaminergic structures of nigrostriatal and mesolimbic systems. (**A**–**D**) IL-6-immunoreactive (yellow arrows), cFos-immunoreactive (green arrows), and double-labeled IL-6/Fos-immunoreactive (white arrows indicate cells with colocalization) cells in the VTA and SNpc, localized in the ipsilateral-to-stimulation brain hemisphere in the representative rats subjected to SHAM (6-OHDA_SHAM) or DBS-STN (6-OHDA_SHAM). The scale bar is 10 µm. (**E**,**F**) Percentages of labeled cells in both brain hemispheres (IPSI, CONTRA) in VTA and SNpc. Data are means ± SEM. Explanation: * *p* < 0.05, ** *p* < 0.01, differences vs. 6_OHDA_SHAM; Tukey HSD after ANOVAs.

The immunofluorescence study showed that the number of IL-6+- and cFos+-containing cells in SNpc and VTA dopaminergic regions in the hemisphere ipsilateral to a lesion were affected by DBS-STN applied in the 6-OHDA-induced model of PD (Figure 4A–F). The Tukey post-hoc test after the one-way ANOVA confirmed elevated percentages of cFos–positive cells in the VTA (F (3.23) = 3.544; *p* < 0.05; Figure 4E) and SNpc (F (3.23) = 3.804; *p* < 0.05; Figure 4F) located in the ipsilateral hemisphere in comparison to the percentages of cells observed in the same hemisphere of 6-OHDA_SHAM. In the same brain structures, the percentages of IL-6-positive labeled cells were reduced (F (3.23) = 4.238; *p* < 0.05 and F (3.23) = 3.804; *p* < 0.05; Figure 4E,F, Tukey post-hoc). Interestingly, the percentage of double-labeled cells (Fos+/IL-6) after DBS-STN was reduced only in the SNpc (F (3.23) = 3.566; *p* < 0.05, Tukey post-hoc).

### 2.5. DBS-STN Affects Plasma Corticosterone Concentration and Peripheral Blood Leukocyte Number

**Figure 5:**
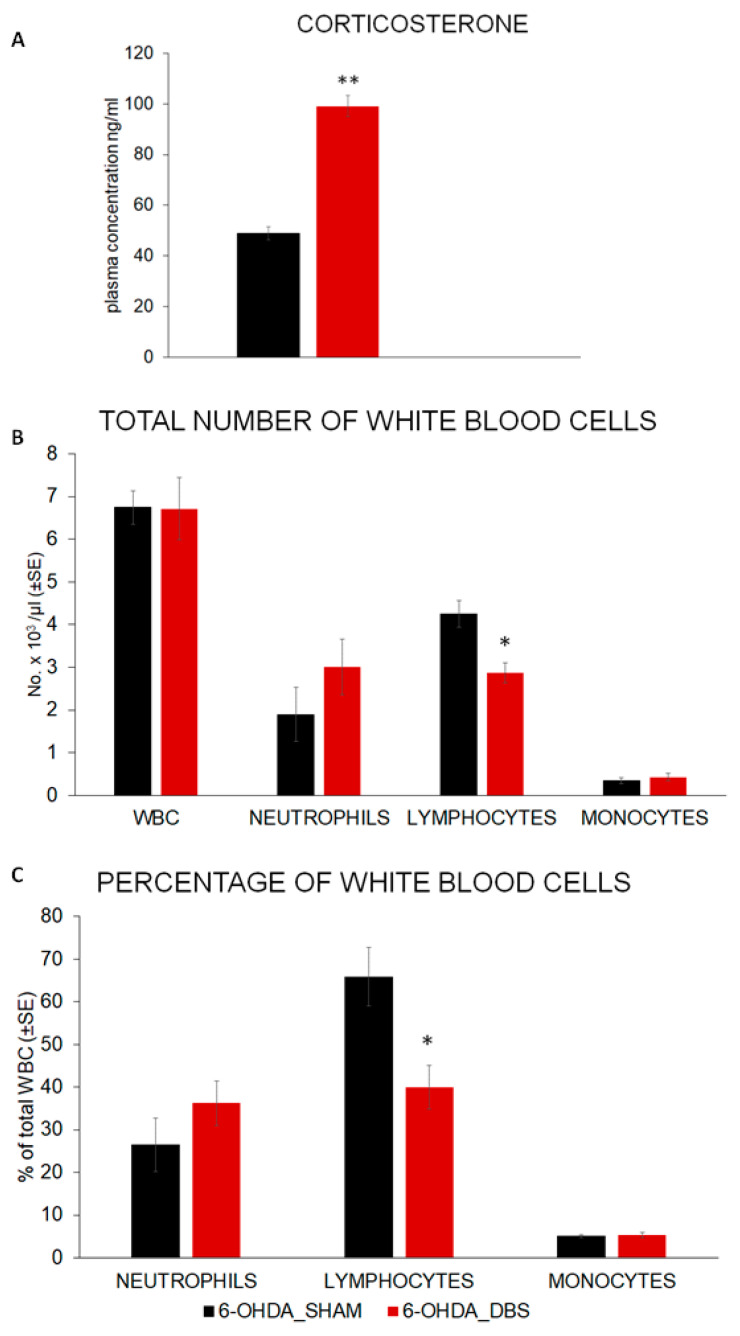
Effect of DBS-STN on plasma corticosterone concentration (**A**), peripheral blood leukocyte percentage (**B**), and number (**C**) in the DBS-STN (6-OHDA_DBS, *n* = 6)- or SHAM (6-OHDA_SHAM, *n* = 6)-stimulated rats within model of early PD. Data are means ± SEM. Explanation: * *p* < 0.05, ** *p* < 0.01 differences vs. 6_OHDA_SHAM (Mann–Whitney U tests).

The Mann–Whitney U test showed that the plasma corticosterone concentration was elevated after DBS-STN applied during neurodegeneration in the nigrostriatal system in rats (U = 2.282, *p* < 0.01; Figure 5A). In parallel, the number (U = 2.419, *p* < 0.05; Figure 5B) and percentage (U = 2.096, *p* < 0.05; Figure 5C) of lymphocytes in peripheral blood decreased in 6-OHDA_DBS rats while other subsets of blood leukocyte numbers did not change.

## 3. Discussion

The present study indicated that DBS-STN, when applied simultaneously with the destruction of dopaminergic cells in the SNpc, resulted in an increase in motivation for food and had beneficial effects on forelimb motor skills. In addition, anxiolytic effects of DBS-STN were observed in our study. Interestingly, behavioral effects of DBS-STN occurred concurrently with an elevated plasma level of corticosterone and a decreased number of lymphocytes in peripheral blood. In parallel, both the reduced percentage of IL-6-labeled cells in the nigrostriatal and mesolimbic (SNpc and VTA) systems as well as activated cFos-labeled cells in these systems were confirmed.

The neurotoxin 6-OHDA administered to the SNpc induced neuronal death mainly in the anterior part of the SNpc. During the Vermicelli test, atypical behaviors characteristic of the complete abolition of transmission in the nigrostriatal system were not observed [35]. Moreover, the effect of DBS-STN on the motor efficiency of the forelimbs in the VHT test was noted. Additionally, the application of DBS-STN for seven consecutive days resulted in a reduction in the time required to eat a piece of pasta as measured during the test.

Rats with partial 6-OHDA lesions in the nigrostriatal system displayed motivation deficits in an operant task [36]. SNpc-lesioned animals also displayed apathy- and depression-like symptoms, including lower preference for food-paired places, increased immobility rates in the forced swim test, and reduced time engaging in social interactions [37]. Partial nigrostriatal denervation has been repeatedly found to spare motor performance while producing motivational impairments in operant tests [38,39]. The most recent study conducted by Pasquerea and Turner [40] demonstrated that in monkeys, STN neurons play a role in feeding behavior and regulate food intake. In mice, the overall activity of STN neurons was enhanced in response to food consumption depending on the size, valence, and palatability of food and the physiological status of the animals [41]. Notably, in their investigation, the optical stimulation of STN neurons decreased food intake whereas the inhibition of STN neurons enhanced food consumption [41]. In conclusion, the authors suggest that the physiological mechanism underlying the weight gain following DBS-STN could involve increasing food intake through the inhibition of STN neurons. Studies by Winter et al. [42] and Klavir et al. [43] proved that in rodents, DBS-STN improves perseverative and compulsive-like behavior. These results are as per our observations. The DBS-STN influenced the motivation for food consumption, which was reflected in shortening the time of eating in the VHT. 

The STN is functionally connected with the VTA and hypothalamus [8]. The mechanism of changes in motivation to eat that were observed in our study may have been related to the STN’s connections with the VTA, a crucial structure of the mesolimbic system. We found that the percentages of cFos-positive neurons in the VTA and SNpc increased after DBS-STN was applied in rats during neurodegeneration compared to sham-stimulated animals. Surprisingly, the study by Wade and collaborators [44], which assessed cFos expression in the mesolimbic system, showed a decreased number of cFos-positive cells. The likely cause for the divergences in the obtained results is probably associated with experimental conditions. Wade et al. [44] applied bilateral DBS-STN in naïve rats (without the PD model) while in our study, the partial dopamine-depleted rats and unilateral DBS-STN were used. In a stable rat model of PD, DBS-STN induces nigrostriatal dopaminergic plasticity [45]. After one week of stimulation, DBS-STN rats exhibited a 3.5-fold increase in the number of TH+ neurons within the SNpc, but not in the VTA, compared to sham controls. There was no difference in basal cell activity, as indicated by cFos expression, in both midbrain dopaminergic systems. In naïve and partially dopamine-depleted rats, DBS-STN increased striatal dopamine efflux and metabolism [46,47,48]. In addition, some studies implicated that DBS-STN’s effects on the cFos marker are extremely time-dependent [49,50,51]. Hence, the outcomes of our study may differ from the aforementioned results.

In addition to the cFos marker, we assessed the percentages of IL-6-labeled cells in both the VTA and SNpc as indicators of neuroinflammation, which arises following the injection of 6-hydroxydopamine into the nigrostriatal system [52]. A distinct association between IL-6 and PD was, for the first time, indicated in the study of Mogi et al. [53]. The immunoreactivity of IL-6 in the SNpc and striatum in PD was prominently higher than that in the control group. Since then, many studies have been conducted that have highlighted the diverse role of IL-6 in PD pathophysiology (for revival, see [54]). In our study, the expression of IL-6-positive cells in the SNpc and VTA was reduced after the application of DBS-STN in the 6-OHDA model of PD. These results are similar to the last study of Pinheiro Campos [34]. They found that 6-OHDA-lesioned rats exhibited increased immunoreactivity of Iba-1 and increased expression of CX3CL1, TNF-α, IL-1β, IL-6, and IFN-γ. After five sessions of DBS-STN treatment, the inhibition of 6-OHDA-induced striatal cytokine expression was observed. In an excellent study by Chen and collaborators [55], similar effects were noted. They found suppressed microglial activation and nuclear factor-κB expression, a decrease in the levels of the pro-inflammatory cytokines interleukin (IL)-1β and IL-6, and an increase in the expression of the anti-inflammatory cytokine IL-4. Additionally, there were downregulations of the IL-1 receptor, extracellular signal-regulated kinase (ERK), and cleaved-caspase3 in the SNpc in rats within a PD model subjected to DBS-STN. The anti-inflammatory effect of DBS-STN has been confirmed in our studies and may promote the increased motivation to eat as a result of the VHT. 

The brain cytokine IL-6 supports healthy weight maintenance in a normal physiological state [56]. The peripheral level of IL-6 is increased in obesity; however, these changes do not correspond with IL-6 levels in the brain, which, instead, are reduced [56]. The interesting study by Mishra [57] indicates that in rats, the infusion of IL-6 to the lateral parabrachial nucleus (lPBN) and paraventricular hypothalamus (PVN) leads to chow intake reduction on a diet that is rich in both fats and sugars. Unfortunately, in this project, the concentration of IL-6 in rat plasma was not measured. However, in our previous studies, we demonstrated that DBS-STN, when applied in unilaterally completely dopamine-depleted rats, reduces plasma IL-6 concentrations, likely through a corticosterone-dependent mechanism [30]. IL-6 can induce the release of corticosterone either directly, by stimulating corticotropin-releasing hormone (CRH)-synthesizing neurons in the PVN, or indirectly, through the stimulation of the production of prostaglandin E2 in perivascular cells [58]. In addition, IL-6 was shown to act directly on anterior pituitary cells as well as in adrenal glands, leading to the synthesis of adrenocorticotropic hormone (ACTH) and glucocorticoids (GC), respectively [59]. Indeed, in our study, the plasma corticosterone concentration was increased after DBS-STN stimulation in rats with early models of PD. This effect was similar to those previously obtained [30] and observed in clinical studies [26]. The anti-inflammatory properties of corticosteroids are often used for the alleviation of fatigue and a loss of appetite in patients with advanced cancer [59,60,61,62]. During acute stress, appetite is typically suppressed [63]. Chronic stress generally promotes the wanting, seeking, and intake of palatable high-fat and energy-dense foods [64]. Stress, particularly chronic stress, has been linked with obesity and weight gain in several but not all studies [65,66]. The effects of chronic stress on food intake and weight may be related to disruptions in the HPA axis. HPA axis activation results in the secretion of cortisol, a glucocorticoid that stimulates appetite and increases the intake of highly palatable foods [67]. A physiologically relevant systemic injection of corticosterone induced an increase in the dopamine transient amplitude and duration in the mesolimbic system [68]. 

Several lines of evidence indicate that corticosterone is an anxiety level modulator [69]. Furthermore, the anxiolytic effect of corticosterone is concomitant with the prevention of impairment in social interaction [70]. In rodents, a single administration of a high dose of corticosterone 1 h after acute and severe stress protects against the anxiogenic and depressive effects of immobilization [71]. Corticosterone administration after cued fear conditioning also suppresses fear-potentiated anxiety as measured by the EPM one week after the administration [72]. Blood sample analysis has revealed that a reduced secretion of corticosterone under basal conditions predicts greater anxiety and avoidance responses, correlating with a decreased corticosterone response to subsequent stressors [73]. In our study, we confirmed that DBS-STN significantly increased the plasma corticosterone levels in rats with early models of PD. We suggest that an elevated corticosterone level promotes exploratory behavior on the open arms of the EPM maze. The anxiolytic effect of DBS-STN in rodent models of PD has been previously described by Faggiani and collaborators [74]. Acute DBS-STN markedly reversed locomotor deficits and anxiety behavior in animals with bilateral DA depletion. 

The influence of stress hormones on peripheral lymphocyte distributions is well documented (for revival, see [75]). In our study, the elevated level of plasma corticosterone after DBS-STN in rats resulted in a reduction in both the percentage and number of peripheral blood lymphocytes. There is growing evidence supporting the role of peripheral immunity in PD patients, including more rapid PD progression in the presence of a pro-inflammatory cytokine profile in the blood [76], a Th1-biased CD4+ T cell profile [77,78], and an altered CD8+ T cell profile, with increased activation and reduced senescence markers [79]. Previously [30], we demonstrated that DBS-STN, when applied in a model of the advanced stage of PD in rats, decreased the percentage of B and CD4+ T lymphocytes in peripheral blood. In this study, the overall effects were similar; unfortunately, the study did not include lymphocyte phenotyping.

Our study was limited regarding its low number of animals per group due to the very high experimental effort involved in such cohorts. We tried to minimize this limitation by using closely matched groups according to their VHT behavior. It is relatively difficult to train rats in the VHT test towards the constant food intake pattern, so finally, only six rats per group were chosen for further experiments. Given the limited number of rats in each group and the deviation of the results from a normal distribution, our primary approach for statistical analysis involved the use of non-parametric tests such as the Kruskal–Wallis and Mann–Whitney U tests. Both abovementioned tests enable the statistical evaluation of results in the absence of heterogeneity of variance to test an experimental hypothesis [80]. Nevertheless, the statistical power of non-parametric tests is typically lower than that of traditional ones; hence, the substantiation of the acquired results on a larger group of animals is required. 

The other limitation of our study was that DBS-STN is typically applied in advanced stages of PD to alleviate motor symptoms, and the timeline of our study did not align with this clinical practice. In our study, the 6-OHDA was administered into the SNpc 24 h prior to the first DBS-STN session. In consideration of prior studies, the motor symptoms are conventionally evaluated no earlier than 3 weeks after the neurotoxin administration [81,82,83], and in rats, the DBS-STN procedure moves on after such behavioral screening [45]. The last study by Slézia and collaborators showed that mild motor impairments, characterized by a dissociation of explorative horizontal locomotion on the open field and motor coordination on the rotarod, are first detectable one week after a striatal 6-OHDA injection [84]. Although neurodegeneration commences earlier after the administration of 6-OHDA into the SNpc, stable observable motor symptoms manifest after three weeks [85]. For this reason, the screening for characteristics of motor deficits (i.e., methamphetamine-induced rotation, rotarod, cylinder test, open-field test) was not performed in our study. 

The main effect of DBS-STN obtained in this study has not been shown so far, and we suggest that it may be caused by anti-inflammatory properties of stimulation on the mesolimbic and nigrostriatal systems. In this study, we demonstrated that the application of DBS-STN during neurodegeneration reduced the percentages of IL-6 labeled cells in the VTA and SNpc and increased the percentage of cFos-positive (activated) neurons in the VTA. The peripheral effects of DBS-STN included an increase in corticosterone levels and a decrease in the number of peripheral blood lymphocytes. 

## 4. Materials and Methods

### 4.1. Animals

Male Wistar rats (*n* = 12), weighing 280–300 g at the time of surgery, were used for the experiments. Animals were kept in standard housing conditions with ad libitum access to standard rat diet (Labofeed B standard, Morawski) and water. Rats were handled on a daily basis to minimize stress caused during experimental procedures. Animals were split into groups:

- 6-OHDA_DBS-group—DBS-STN after intranigral injection of 6-hydroxydopamine (6-OHDA) to mimic PD (*n* = 6),
- 6-OHDA_SHAM group—unstimulated rats with intranigral injection of 6-OHDA (*n* = 6) as control group.

**Figure 6:**
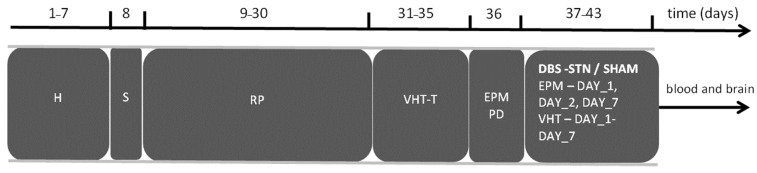
Schematic diagram of experimental timeline in 6-OHDA_SHAM (*n* = 6) and 6-OHDA_DBS (*n* = 6) groups. Explanations—H: handling; S: stereotactic electrode implantation into right subthalamic nucleus (STN; AP = −3.6 mm; L = −2.6 mm; D = 8.0 mm) and guiding of cannula into substantia nigra pars compacta (SNpc; AP = −5.3 mm; L = −2.4 mm; D = −7.5 mm) according to [86] coordinates. RP: recovery period in home cage; VHT-T: training for pasta eating in the presence of the experimenter prior to Vermicelli handling test; EPM: elevated maze test before PD model induction and after DBS-STN or SHAM stimulation (1st, 2nd, and 7th day of stimulation); PD: 12 μg of 6 hydroxydopamine dissolved in 4 μL 0.9% NaCl with ascorbic acid microinfusion into the right SNpc. DBS-STN procedures were applied daily for seven days, for 1 h each, with continuous electrical high-frequency stimulation of subthalamic nucleus (current frequency: 130 Hz, pulse width: 60 µs, stimulation intensity: 70–220 µA) in DBS-STN group; VHT: Vermicelli handling test was performed every day after DBS-STN or SHAM stimulation (1st to 7th day of stimulation). Blood and brain collection: blood samples were collected via cardiac puncture.

The procedures applied in both groups are presented in Figure 6. All procedures were approved by the Local Ethical Committee for the Care and Use of Laboratory Animals in Bydgoszcz, Poland 36/2015 and were in accordance with the EU Directive 2010/63/EU. 

### 4.2. Stereotaxic Implantation of Cannulae into the SNpc and Stimulating Electrode into the STN

The rats were anesthetized with 1.5–2.5% isoflurane (airflow: 0.5 L per min) using an isoflurane pump (Bitmos OXY 6000, Bitmos GmbH Düsseldorf, Düsseldorf, Germany), and analgetic butorphanol 2.0 mg/kg i.s. (Butomidor, Richter Pharma, Wels, Austria) was administered. A stainless-steel guide cannula (22 GA, 9 mm long; Plastic One, Roanoke, VA, USA) was implanted 1 mm above the SNpc according to the stereotaxic coordinates: −5.3 mm anterior to the bregma, −2.4 mm lateral to the midline, and 6.4 mm below the skull surface [86] using stereotactic apparatus (Kopf Instruments, Tujunga, CA, USA). The cannulae were permanently anchored to four stainless-steel skull screws with dental acrylic (Duracryl, Spofa Dental a.s., Jičín, Czech Republic). After cannula implantation, the monopolar stainless-steel electrode (0.2 mm diameter, Plastic One, Eschweiler, Germany) in the right STN was implanted. The stereotaxic coordinates from STN were as follows: AP −3.6 mm; L −2.6 mm; D −8.0 mm [86]. Rats postoperatively received antibiotic solution (Penicillin procaine, Polfa, Poland). After surgery, the animals were transferred to a warm room, where they stayed until regaining consciousness. The behavioral testsinjection and stimulation procedures started after a 21 day recovery period from the surgery (Figure 6).

### 4.3. Unilateral Model of Partial Nigral Depletion

During the infusion procedure, each animal was held gently by hand. The 6-OHDA (6-hydroxydopamine HCl, Sigma–Aldrich, Poznań, Poland) was injected into the right SNpc in a volume of 2 μL (1.5 μg/μL dissolved in 0.9% NaCl containing 0.1% ascorbic acid). Injection was performed using a microinfusion pump (Legato 100—Series Syringe Pump, KD SCIENTIFIC, Holliston, MA, USA) and a Hamilton syringe (10 μL) connected via polyethylene tube to an injection cannula (28 GA, 10 mm long, Plastic One, USA), which was placed into the guide cannula. Injection rate was 0.5 μL/min, and the cannula was left in place for additional 5 min after injection to allow for diffusion into tissue. To protect noradrenergic neurons from damage, animals received an intraperitoneal injection of the noradrenaline reuptake inhibitor desipramine (25 mg/kg, Sigma-Aldrich, Poznań, Poland) 30 min prior to neurotoxin injection [30].

### 4.4. Deep-Brain Stimulation of Subthalamic Nucleus

After a three-week recovery period, rats were habituated to DBS-STN conditions for three consecutive days. Animals were taken from their home cages and placed in transparent plexiglas testing cylinders (gauge: 30 cm; height: 40 cm) and were allowed to explore the boxes for 30 min. DBS-STN was performed on the next day after 6-OHDA injection in accordance with method that we described previously [30]. Within the first 5 min of screening stimulation, the behavioral effects of increasing the stimulation intensity from 0 to 150 μA were examined in individual animals. During this procedure, contralateral torsion of the head or dyskinetic movements of the contralateral forelimb were observed. A range of current intensity was set at 70–220 µA, values just below provocation of dyskinetic movements. The following day, current intensity was applied continuously for 1 h throughout the DBS-STN procedure. The stimulation duration and parameters were determined based on the method described by Salin et al. [13]. Stimuli were delivered by a stimulator unit (215/T, Hugo Sachs Elektronik D7806 March F.R., Breisgau, Germany) that provided rectangular pulses. The frequency was set at 130 Hz and the pulse width at 60 µs all over the stimulation period for all the stimulated animals. Sham-stimulated controls underwent the same procedure excluding current flow.

### 4.5. Elevated Plus Maze (EPM)

This test was performed immediately before 6-OHDA microinjection (as baseline), one day after 6-OHDA administration, and on 7th day of HFS-STN stimulation (immediately after end of HFS-STN procedure) (see Figure 6). The EPM consisted of two open arms (10 cm in width and 50 cm in length) and two enclosed arms (10 cm in width, 50 cm in length and 40 cm in height), elevated 50 cm above the floor. The EPM was cleaned with 70% ethanol before the start of every trial. After disconnecting the stimulation cable, the rat was put on the center square of the EPM. Rat was placed in the maze, always in the same position (heading towards the open end of the maze). The animal was allowed to explore the EPM for 5 min and movement was recorded using a video camera (Ikegami, Ikegami Electronics, Neuss, Germany). A video camera was positioned approximately 250 cm over the center of the maze and connected to a video-tracking digitizing device (EthoVision XT10, Noldus, Wageningen, The Netherlands). Registered and analyzed reactions included time spent in open/closed arms and in the center of the maze and number of entries into open/closed arms and to the center of the maze, as we previously described [87]. In the results section, we have presented number of entries into open/closed arms of maze and time spent in each one of the arms.

### 4.6. Vermicelli Handling Tests (VHT)

VHT testing began three weeks after surgery. Procedures for the VHT were based on those developed by Allred et al. [35]. To allow familiarization with the testing environment and reduce the possibility of neophobia to the vermicelli, rats were given three acclimatization sessions with seven strips of 7 cm vermicelli, which were inserted into the cage after the rat in question had finished eating the previous piece (Pastifico Fabianelli S.p.A, Castiglion Fiorentino, Italy). In the final acclimatization session, all rats consumed all pasta strips within 20 min. The testing conditions were the same in both groups. The VHT was conducted in stimulation room. After end of DBS-STN or sham procedure, rat was immediately placed in his home cage and the test was started. High-definition video cameras (Canon IXUS 145) were placed on either side of the cage to capture the rats’ movements within the cage. The vermicelli was marked at 1 cm intervals with food coloring to increase visualization of pasta movement. For both groups, testing was completed before 6-OHDA injection, one day after, and daily during seven days of DBS-STN procedure. Videos were scored by an experimenter blind to the subjects’ lesion condition and testing timepoint. The initial scoring was based on the criteria described by Allred et al. [35]. Videos were viewed in high resolution at slow speed (×0.5). The first three video-recorded pieces of vermicelli consumed by each rat were scored; when a piece was not scorable due to being eaten outside the field of view of the cameras, the next viewable piece was scored. The adjustments of each paw were analyzed and reported as the mean number per trial per session (three pasta pieces per session). The total number of adjustments of each paw was counted per trial (TO) together with the number of touches of paw as grasp (OD). Then, the Vermicelli asymmetry ratio (VAR) was calculated according to the formula below: VAR = (OD/TO) × 100. In addition to VAR, the time of manipulation was analyzed and reported as the mean time of eating one vermicelli piece per session.

### 4.7. Blood Collection

Blood samples were collected via heart puncture under 2.5% isoflurane anesthesia (airflow: 0.5 L per min) using an isoflurane pump (Bitmos OXY 6000, Bitmos GmbH, Düsseldorf, Germany) between 09.00 and 10.00 AM and 1 h after the DBS-STN or sham stimulation. The blood samples were divided into two tubes. One of tubes was centrifuged (10 min, 3000× *g*) to obtain fresh plasma without platelets and cells. The supernatant was transferred to Eppendorf tubes, quickly frozen at −70 °C, and stored to analyze plasma CORT. The second part of the sample was tested immediately using automated hematology analyzer (Cell Dyn 3700). Peripheral blood hematological parameters including the total white cell (WBC) number and percentages of lymphocytes (LYM), neutrophils (NEU), and monocytes (MON) were determined in the EDTA_K2 blood as we previously described [30]. 

### 4.8. Plasma Corticosterone Determination

Plasma corticosterone concentrations were measured via radioimmunoassay using a commercially available kit (Rat corticosterone 125I RIA Kit, MP Biomedicals, Santa Ana, CA, USA) and Wizard 1470 gamma counter (Pharmacia-LKB, Helsinki, Finland) in accordance with previously used method [30,87,88]. The minimal detectable dose in this system was 7.7 ng/mL.

### 4.9. Brain Tissue Preparation

One hour after the end of DBS-STN or sham stimulation, rats were euthanized with Morbital (2 mL/kg) and transcardially perfused (via the left ventricle) with 200 mL of 0.9% saline followed by 200 mL of 4% paraformaldehyde in 0.1 M phosphate-buffered saline (PBS).

The brains were removed quickly, postfixed, cryoprotected in a 30% sucrose solution in PBS, and then frozen and kept at −70 °C until cryostat sectioning (CM 1850, Leica Biosystems, Wetzlar, Germany). Coronal 20 µm thick sections containing the VTA and SNpc (5.04 mm posterior to the bregma) were chosen for the double immunofluorescence method for IL-6 and cFos protein detection. For tyrosine hydroxylase and Nissl staining, the coronal 30 μm thick tissue sections were cut at the level of the 1.92 mm anterior to the bregma (according to [86]) containing CPu, −5.04 mm anterior to the bregma containing SNpc, and −3.72 mm anterior to the bregma containing STN (for Nissl staining).

### 4.10. Immunohistochemistry for TH-Expression

To determine the loss of dopaminergic neurons in the SNpc, we used immunohistochemical staining of tyrosine hydroxylase (TH) as previously described (for details, see [89]). Briefly, prior to all the immunohistochemical stages, the sections were rinsed several times in PBS, then incubated in 0.3% hydrogen peroxide in PBS for 10 min at room temperature and blocked for 45 min with a solution of 1% Bovine Serum Albumin (BSA) (BioChemika, Fluka, Buchs, Switzerland) and 0.3% Triton X-100 in PBS at room temperature for the effective reduction of nonspecific binding. Next, the sections were incubated with a polyclonal rabbit anti-TH antibody (Novus Biologicals, Centennial, CO, USA, NB300-109) at a dilution of 1:1500 (diluted in PBS containing 0.3% TritonX-100 and 3% Normal Goat Serum (NGS, Sigma-Aldrich, Poznań, Poland)) at 4 °C for 3 days. For light visualization, after a 30 min incubation with biotinylated goat anti-rabbit IgG (a dilution of 1:200; Vector, Charlotte, NC, USA) in PBS containing 0.02% sodium azide and 0.3% Triton X-100 at room temperature, the sections were rinsed with PBS + Triton and incubated with avidin–biotin peroxidase complex (ABC) (a dilution of 1:100 in PBS; Vector Elite Kit, Burlingame, CA, USA) for 1 h at room temperature. Washed in PBS, the sections were incubated in 40 mL Tris buffer (pH 7.6) (BioChemika, Fluka) containing 30 mg of diaminobenzidine tetrahydrochloride (DAB) (Sigma-Aldrich, Poznań, Poland). After a few minutes, the sections were incubated with 30% hydrogen peroxide (H_2_O_2_) (solution 90 μL H_2_O_2_/10 mL PBS, Eurochem BGD, Tarnow, Poland) and allowed to react for 15–20 min. The reaction was controlled and stopped in Tris buffer when the TH-immunoreactive cells turned brown. The tissue sections were placed on slides, air dried, and, after dehydration with ethanol, mounted with DPX (DPX Mountant for histology, Sigma-Aldrich, Poznań, Poland).

### 4.11. Immunofluorescence for IL-6 and cFos Protein Colocalization

Immunofluorescence processing for IL-6 and cFos protein colocalization (IL-6/cFos+ cells) was performed in accordance with the method that we previously described [90] with some modifications. After unmasking the antigen in 0.01 M citrate buffer (pH 6.0, 74 °C for 40 min), the sections were blocked in 5% bovine serum albumin (BSA) in PBS with 0.02% Triton X for 2 h. Then sections were incubated for 48 h at 4 °C in a cocktail of primary antibodies containing rabbit anti IL-6 IgG (Abcam, Anti-IL-6 antibody EPR23819-11; dilution 1:300) and mouse anti-cFos IgG (Santa Cruz Biotechnology sc-166940, dilution 1:300) diluted in PBS with 0.2% Triton X. Following multiple rinses in 0.05 M Tris buffer (pH 7.4), the sections were incubated for 2 h in a cocktail of secondary antibodies conjugated with fluorochromes containing Alexa Fluor 488 goat anti-rabbit IgG (Invitrogen, Carlsbad, CA, USA, A11034, dilution 1:400) and Alexa Fluor 546 goat anti-mouse IgG (Invitrogen, A11030, dilution 1:400). Dilutions of primary and secondary antibodies were established according to the producers’ recommendations and our preliminary methodological trials. The test for the specificity of an antibody involved (negative control) was performed by omitting the primary antibodies. All stages of immunofluorescence were identical, as described previously, except for the first incubation: sections were placed in PBS with 0.2% Triton X for 48 h without primary antibodies.

### 4.12. Microscopic Analysis

The counting of labeled cells’ TH+ cell bodies was performed in three sections of the SNpc (−5.04 mm from Bregma). For TH+ cell body counting, a light microscope, Leica DC300 (magnification 10 × 10), and Leica Qwin Software (Serial No. 4358) were used. Then, the mean value of TH+ neurons from each animal was expressed as a percentage of cell loss on the lesioned side compared to non-lesioned side. 

Immunofluorescence staining sections were viewed at 20 × 10 magnification by an investigator blinded to the assignment of treatment groups. Slices were examined under a Zeiss Axio Scope.A1 fluorescent microscope with camera (AxioCam) and AxioVision Rel.4.8 software. To quantify immunoreactivity, square test area (0.01 mm^2^) was chosen randomly from representative sections within each tested brain area. The borders of the brain structures were determined based on the Paxinos and Watson atlas [25]. In every test area, the number of colocalized IL-6/cFos positive cells (IL-6/cFos+) and the numbers of only IL-6 positive (IL-6+) and only cFos-positive (cFos+) cells were counted. The percentage of double IL-6/cFos immunofluorescent cells (IL-6/cFos+) as part of all immunofluorescent (only IL-6-positive + only cFos-positive + IL-6/cFos double-labeled) cells was estimated.

To determine the location of the stimulating electrode, we used the Nissl staining. The selected sections were placed on slides, stained with Cresyl violet (Sigma-Aldrich, Poznań, Poland), dehydrated, and finally mounted with DPX (Sigma-Aldrich, Poznań, Poland). Animals showing a misplaced electrode were not included in the experimental groups presented above.

### 4.13. Statistical Analysis

The statistical analyses were performed using IBM SPSS Statistics 27.0 and the level of significance was set at *p* ≤ 0.05. The statistical evaluation of the mean TH+ cells in the SNpc, cFos+, and IL-6+ were assessed using one-way ANOVA. The main factor was experimental group (DBS or SHAM). Additionally in each group, a division into two brain hemispheres was applied (contra- and ipsilateral to the stimulation), which resulted in four independent groups of outcomes. The differences in the means were further analyzed with Tukey’s HSD post-hoc. The behavioral data were analyzed using Kruskal–Wallis non-parametric tests and post-hoc Dunn tests. The hematological data and data from plasma corticosterone levels were analyzed using U-Man Whitney tests. All data were expressed as means ± SEM.

## Data Availability

The data presented in this study are available on request from the corresponding author.
